# Concurrent Nerve and Tendon Transfers in a Tetraplegic Patient: Long-Term Functional Recovery and Late Hardware-Associated Flexor Pollicis Longus Rupture

**DOI:** 10.7759/cureus.107227

**Published:** 2026-04-17

**Authors:** Pranav Bingi, Yehuda A Masturov, Loren Shamalov, Matthew S Wilson, Andrew J Miller, Adam B Strohl

**Affiliations:** 1 Sidney Kimmel Medical College, Thomas Jefferson University, Philadelphia, USA; 2 Orthopedic Surgery, Philadelphia Hand to Shoulder Center, Philadelphia, USA; 3 School of Medicine, The City University of New York, New York City, USA

**Keywords:** nerve transfer, spinal cord injury rehabilitation, tendon transfer, tetraplegia, upper extremity reconstruction

## Abstract

Tetraplegia is a significant impairment of mobility in all four limbs, often following a cervical spinal cord injury. Recent studies in the management of tetraplegia describe the use of nerve transfer in addition to tendon transfer for upper extremity rehabilitation. While the popularity of these techniques is growing, there are limited studies describing the long-term outcomes and functional recovery following such procedures.

This case describes a patient who sustained a C7 spinal cord injury, right olecranon fracture, and left distal radius fracture following a fall. Surgeons performed open reduction and internal fixation for the extremity fractures. The C7 injury resulted in loss of digital flexion and extension as well as significant weakness in his right elbow. Two years after the initial injury, surgeons performed a nerve and tendon transfer procedure, with significant postoperative improvements in right elbow flexion and bilateral pinch and grasp functions. Four years after the initial trauma, the patient returned with a recent loss of thumb interphalangeal flexion caused by a left flexor pollicis longus rupture secondary to hardware from the distal radius fixation. His subsequent treatment included surgical intervention for hardware removal and intercalated autograft.

This case serves to guide the future surgical management of tetraplegia with insight into the complications and long-term outcomes of concurrent nerve and tendon transfer.

## Introduction

Cervical spine injury results in a loss of mobility in all four extremities, resulting in tetraplegia. Tetraplegia poses a significant challenge for patients, greatly impacting their ability to perform activities of daily living and fulfill life roles [1]. Reconstruction of the upper extremity with tendon transfer has long served as a treatment option for tetraplegia, serving to improve hand, wrist, and elbow functions as well as patient satisfaction [1].

Recent studies support nerve transfer as a viable alternative to tendon transfer, demonstrating comparable postoperative upper extremity strength and effectively improving motion of the upper extremity [2]. Use of a combination of nerve and tendon transfers has also gained popularity, allowing for improved versatility in hand motion and contributing to long-lasting improvements in functionality [3].

This case report presents the surgical management and long-term outcomes of a hybrid approach with a nerve and tendon transfer procedure. The case also includes a postoperative complication which resulted in a tendon rupture and required a secondary procedure. Understanding the long-term management of tetraplegia in the setting of potential complications from previous procedures is critical to improving patient outcomes and guiding clinical judgment. Furthermore, salvaging the already completed and functioning transfers is more complicated but paramount to maintaining function in this patient population.

## Case presentation

A man in his 30s, with an unremarkable social and family background, presented to the outpatient clinic for C7 spinal cord injury with resultant tetraplegia secondary to a fall two years prior. Prior surgical management involved anterior-posterior cervical spinal fusion with open reduction and internal fixation performed for concurrent right (R) olecranon and left (L) distal radius fractures.

Physical examination revealed good distal capillary refill and sensation to light touch but demonstrated poor mobility. The patient exhibited reduced wrist flexion (3/5 R and 4/5 L) and elbow extension (3/5 R and 3/5 L) along with no active extension or flexion in the fingers or thumbs. With his R hand, he was 3 cm shy of being able to form a full grip, with thumb brace application improving pinch on the L side. The rest of the upper extremity function was maintained: bilateral shoulder abduction, adduction, and internal and external rotation, bilateral elbow flexion, bilateral forearm supination and pronation, and bilateral wrist extension.

**Figure 1:**
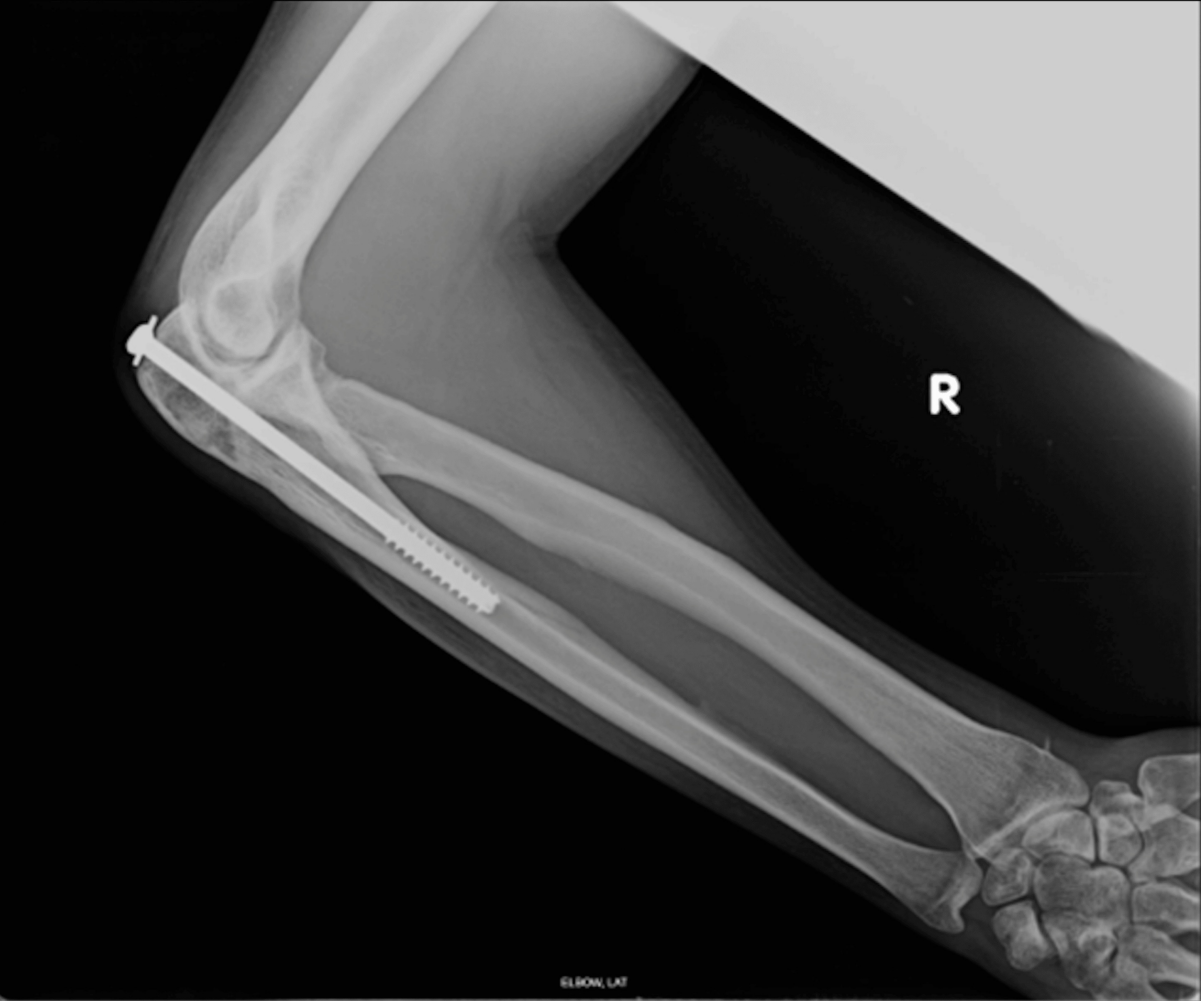
Right lateral elbow X-ray demonstrating retained olecranon screw

Prior to surgical planning, appropriate radiographic imaging was ordered (Figure 1). Radiographic imaging revealed a right olecranon screw secondary to open reduction and internal fixation performed after the initial injury. To ensure consistent goals of care, a thorough review of treatment goals and current functionality was conducted.

Treatment

The main treatment goals for this tetraplegic patient were to restore flexion in the left digits, restore flexion and extension in the right digits, and strengthen right elbow extension. The patient elected to undergo a bilateral tendon and nerve transfer procedure, including right biceps to triceps tendon transfer, right brachioradialis (BR) to flexor pollicis longus (FPL) tendon transfer, right split FPL to extensor pollicis longus (EPL) procedure, right extensor carpi radialis longus (ECRL) to flexor digitorum profundus (FDP) tendon transfer, and left supinator to anterior interosseous nerve (AIN) transfer.

On the left side, donor nerve availability was confirmed with the use of a handheld intraoperative stimulator, with left AIN stimulation resulting in thumb and digit flexion. Two supinator branches were verified to be viable donors and were subsequently transected distally and coapted to the proximal end of the AIN in the forearm.

On the right side, the previously placed olecranon hardware was removed. The biceps was dissected from the surrounding fascia and its tendon subsequently released from the radial tuberosity. The distal end of the biceps tendon was passed and woven through the distal triceps and its tendon. Utilizing the existing olecranon hole, a bone tunnel was constructed through the ulna to dock the distal biceps tendon and complete the biceps to triceps tendon transfer.

The dissection was extended distally to prepare the forearm musculature for tendon transfers. The BR and ECRL tendons were identified and incised from their distal attachments. The ECRL was passed through and secured to the four FDP tendons using a Pulvertaft weave once appropriately tensioned. The BR tendon was transferred to the FPL tendon with a single Pulvertaft. To achieve a stable thumb interphalangeal (IP) joint, a split FPL transfer was performed.

Physical therapy was arranged following surgical intervention to address the extension contracture of the right hand and rehabilitate finger flexion. The patient was counseled on future tendon transfers to regain finger extension.

Outcomes and follow-up

At six months, the patient was able to perform weighted elbow extension (5/5 triceps) for transferring and flex his left index and thumb IP joints (5/5 FPL and FDP) against resistance, confirming successful supinator to AIN transfer. Digital flexion continued to improve as did his functionality.

**Figure 2:**
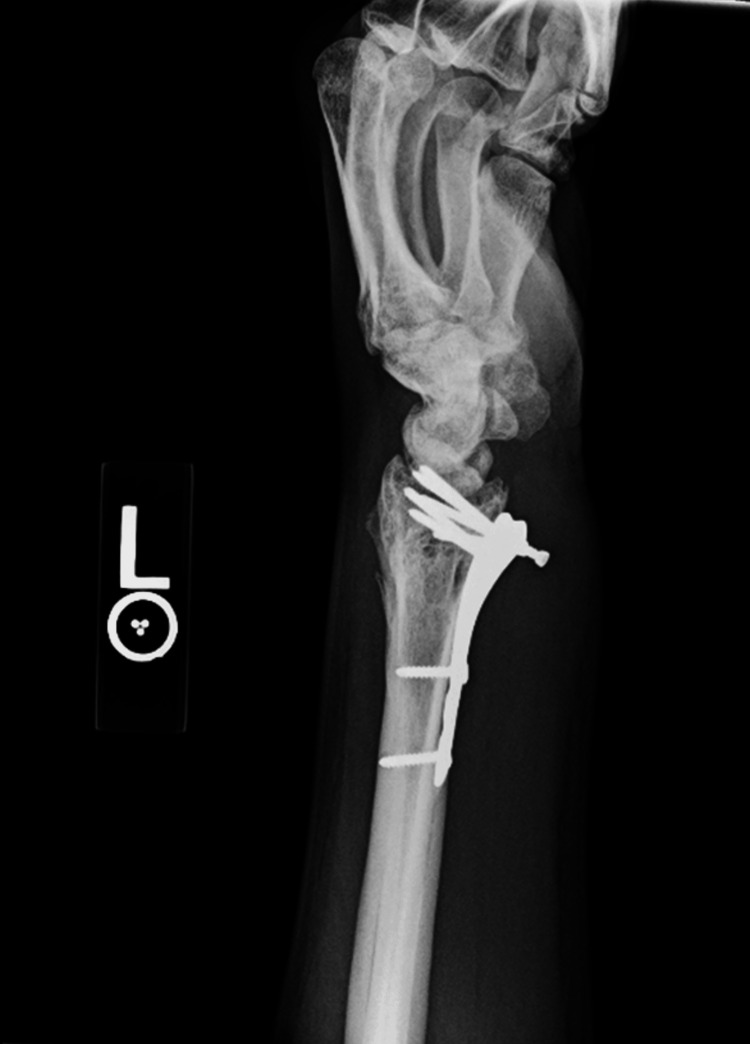
Left lateral wrist X-ray demonstrating volar screw displacement

The patient missed follow-up appointments and returned two years after his last appointment with complaints of loss of function of the left thumb. Physical exam revealed absent left thumb IP flexion. Ultrasound revealed tendon disruption and distal retraction of the FPL tendon consistent with FPL rupture. Radiographic findings discovered retained hardware from about the distal radius with a prominent screw volarly (Figure 2). The team recommended hardware removal with FPL reconstruction to which the patient agreed.

Three months after his initial visit for the left FPL tendon, he underwent tendon transfer surgery. Concurrent FDP rupture of his index finger was also noted. Following the removal of the distal radius hardware, the surgeons opted for tenolysis of the FDP and FDS tendons of the third and fifth digits. Surgeons performed a side-to-side tenodesis tendon transfer between the index finger and long finger FDP tendons to strengthen the ruptured index FDP tendon. To allow for flexion of the thumb, the ring finger FDS tendon was harvested and used as an interpositional graft to reconstruct the FPL tendon.

**Figure 3:**
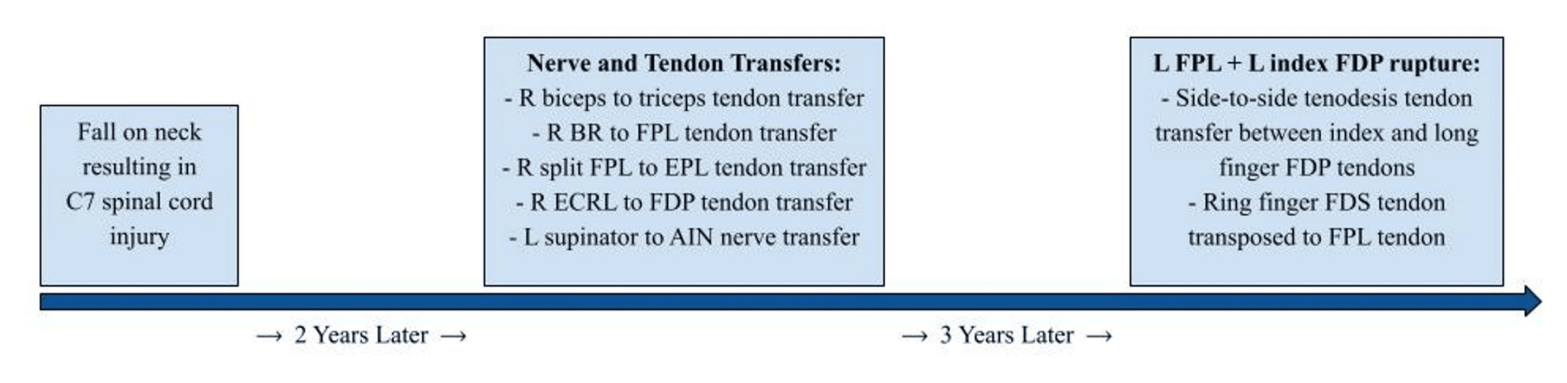
Timeline of injuries and subsequent treatments R: right; BR: brachioradialis; FPL: flexor pollicis longus; EPL: extensor pollicis longus; ECRL: extensor carpi radialis longus; FDP: flexor digitorum profundus; L: left; AIN: anterior interosseous nerve

Twelve days postoperatively, he demonstrated active index and thumb finger flexion while maintaining flexion in the other digits. Four weeks postoperatively, he was able to make a full fist, and by five weeks, he experienced an overall 10-degree increase in metacarpophalangeal (MCP) joint flexion. At 10 weeks postoperatively, all tendon transfers remained intact, and he was advised to discontinue splint wear while continuing physical therapy. He ultimately returned to his pre-rupture functional status. An overview of injuries and treatment regimens is outlined in Figure 3.

## Discussion

​​Surgical restoration of the tetraplegic upper extremity vastly improves quality of life. An individual's International Classification for Surgery of the Hand in Tetraplegia (ICSHT) provides a framework for the surgical procedures available to restore mobility based on donor availability [1].

Although tendon and nerve transfers are both viable options, tendon transfers remain the cornerstone for reanimating the tetraplegic upper extremity [1]. Restoration of elbow extension is achieved with either biceps to triceps or deltoid to triceps tendon transfer. Deltoid to triceps tendon transfer can be utilized in patients with strong deltoids; however, biceps to triceps tendon transfer allows for greater extension against gravity and force generation despite small sacrifices in elbow flexion [4,5].

Nerve transfer surgery has recently garnered popularity in the management of tetraplegic patients. One case report describes the successful nerve transfer of the posterior deltoid branch of the axillary nerve to the triceps long head branch of the radial nerve for the reanimation of elbow extension, the long thoracic nerve to the posterior interosseous nerve (PIN) for finger and thumb extension, and the pronator teres branch of the median nerve to AIN for finger and thumb flexion [2]. Supinator to AIN transfer has also emerged as an effective reconstruction technique to improve pinch and grasp function [3,6].

The patient presented with no movement in his fingers and thumbs bilaterally and significantly reduced right-sided elbow extension movement. As described by the ICSHT, restoration of elbow extension, wrist extension, and pinch function were prioritized [7]. Right biceps to triceps tendon transfer resulted in steady and significant improvement of elbow extension without significant loss of elbow flexion. The procedure's success validates current trends in tetraplegia management which favor biceps to triceps tendon transfer [4,5]. However, it is emphasized that treatment decisions are largely based on a patient's presenting upper extremity function.

To target hand pinch and grasp function, multiple tendon transfers were conducted on the right upper extremity including an ECRL to FDP tendon transfer, a BR to FPL tendon transfer, and a split FPL tendon transfer. In contrast, the patient underwent a supinator to AIN transfer on the left to reanimate thumb and digital flexion [3,6,8]. The restoration of hand pinch and grasp function is one of the first upper extremity movements to utilize a combined nerve and tendon transfer [3]. This case presents concurrent rather than combined use of tendon and nerve transfers to restore the tetraplegic hand.

FPL rupture in the setting of volar plate hardware is a well-documented complication. However, there is a lack of significant research on the impact of such hardware failure in tetraplegic patients [9]. In patients with pre-existing low motor function, FPL rupture may present subclinically, requiring increased vigilance from the care team during surgical planning and in follow-up. The patient recognized the loss of thumb flexion; however, surgeons should ensure that a comprehensive physical examination is performed to identify any secondary complications during follow-up. It is possible that the increased load across the wrist may have further loosened hardware leading to impingement in the setting of a now functioning FPL tendon. This hardware-related complication is a unique phenomenon which can be clinically silent before reanimation using tendon and/or nerve transfers [10].

In addition, the limited tendon donors in this tetraplegic patient limit the options in the surgical management of FPL and index FDP rupture. The cornerstone of FPL reconstruction is either palmaris longus autografting or tendon transfer [10], with both offering similar functional outcomes. The chronicity of symptoms and the attritional nature of the rupture precluded FPL repair. As such, an FPL reconstruction was performed with an intercalated autograft using the FDS to the ring finger. The concurrent index FDP rupture in the setting of ipsilateral supinator to AIN transfer remained another point of contention. Adequate power for the transfers must be maintained for the patient to achieve synchronous finger flexion and grip. The surgeons opted to perform side-to-side tenodesis transfer from the index FDP to long FDP tendons, which resulted in the maintenance of digit flexion.

## Conclusions

Based on the available literature, there is no case describing concurrent tendon and nerve transfers complicated by FPL and FDP rupture in the setting of tetraplegia. By presenting the management of tetraplegia over a five-year period, this study aims to advance current understandings of tetraplegia management and knowledge of specific associated risk factors and complications. This case further demonstrates the importance of interdisciplinary collaboration in the postoperative management of tetraplegia, with patient adherence to physical and occupational therapy playing a major role in the restoration of hand and elbow function. Some limitations include absent objective pre- and postoperative outcome measures, lack of standardized functional scoring, and reliance on subjective measures of improvement. Nonetheless, this case illustrates that surgeons must maintain a high degree of vigilance after nerve and tendon transfer reconstruction. 
